# The association between vital signs at hospital admission and adverse outcomes in patients with COVID-19: a retrospective cohort study

**DOI:** 10.3389/fmed.2025.1602129

**Published:** 2025-07-03

**Authors:** Marat Murzabekov, Niklas Lidströmer, Niklas Borg, Michael Runold, Eric Herlenius, Susanne Rautiainen

**Affiliations:** ^1^Department of Women's and Children's Health, Karolinska Institutet, Stockholm, Sweden; ^2^Astrid Lindgren Children's Hospital, Karolinska University Hospital, Stockholm, Sweden; ^3^Center for Molecular Medicine, Karolinska University Hospital, Solna, Sweden; ^4^Department of Medicine Solna, Karolinska Institutet, Stockholm, Sweden; ^5^Clinical Epidemiology Division, Department of Medicine Solna, Karolinska Institutet, Stockholm, Sweden

**Keywords:** COVID-19, vital signs, respiratory rate, saturation, temperature, clinical decision support systems

## Abstract

**Background:**

Vital sign measurements at hospital admission are used to identify patients at risk for adverse events. However, how vital signs at admission are related to adverse outcomes among COVID-19 patients is not fully characterized.

**Objectives:**

To characterize vital signs at admission and their associations with intensive care unit/intermediate care unit (ICU/IMCU) admission and in-hospital mortality in adult patients with COVID-19.

**Methods:**

This retrospective cohort study included 2,826 adults admitted with COVID-19 to Karolinska University Hospitals, Stockholm, Sweden, between 2 March 2020 and 1 June 2021. The Cox proportional hazards model was used to estimate hazard ratios (HRs) and 95% confidence intervals (CIs) for associations between vital signs at admission and ICU/IMCU admission and in-hospital mortality.

**Results:**

The median age was 62.2 years, and 62.1% were men. Each unit increase in respiratory rate (HR 1.03, 95% CI 1.02–1.05), heart rate (HR 1.01, 95% CI 1.00–1.02), temperature (HR 1.21, 95% CI 1.11–1.32), and each unit decrease in saturation (HR 1.05, 95% CI 1.04–1.06) were associated with ICU/IMCU admission. Respiratory rate (HR 1.04, 95% CI 1.02–1.07) and saturation (HR 1.04, 95% CI 1.02–1.06) were also associated with in-hospital mortality. These associations persisted across pandemic waves.

**Conclusion:**

Respiratory rate and lower saturation at admission were associated with increased ICU/IMCU admission and in-hospital mortality. Our findings suggest that greater emphasis on respiratory rate and oxygen saturation in early warning scores—such as the revised Sequential Organ Failure Assessment (SOFA) score and other sepsis prediction models, to improve risk stratification of viral sepsis, especially in patients with COVID-19.

## Introduction

Since the first case of COVID-19 caused by SARS-CoV-2 infection was reported, rapid progress has been made in understanding the pathogenesis of the disease, therapeutic strategies, and the development of effective vaccines. The severity and characteristics of SARS-CoV-2 infection and COVID-19 vary widely, and most cases require no medical treatment (1, 2).

However, a subset of patients exhibit rapid disease progression, culminating in critical illness characterized by respiratory failure, pulmonary compromise, and the onset of acute respiratory distress syndrome (ARDS). These severe manifestations underscore the multifaceted pathophysiology of COVID-19, implicating dysregulated immune responses, microvascular dysfunction, and aberrant coagulation pathways (3).

The rapid response by the scientific and medical community have led to the development of SARS-CoV-2 vaccines and enhanced understanding of effective treatments, significantly reducing the burden and severity of COVID-19. However, new variants of SARS-CoV-2, such as Delta and Omicron, continue to pose challenges through their increased transmissibility and potential for immune evasion (4), thus further complicating the clinical landscape. Early observations of severe COVID-19 cases have enhanced clinical practices and risk stratification to identify individuals at risk of clinical deterioration and mortality (5, 6). However, there is a need of studies from later stages of the pandemic to ensure that current clinical practices retain their prognostic accuracy given the changing characteristics of the disease. Moreover, COVID-19 can lead to sepsis, a serious, sometimes fatal overreaction of the immune system to an infection. While sepsis is most associated with bacterial infections, it is now clear that viral infections, including COVID-19, can also trigger sepsis, referred to as *viral sepsis*. Sepsis cause ~11 million deaths each year worldwide. Therefore, early recognition of sepsis is critical because timely intervention significantly reduces mortality and improves patient outcomes.

Within emergency departments and other clinical settings, the assessment of physiological vital signs, including temperature, heart rate, blood pressure, respiratory rate and oxygen saturation, is an integral and pivotal tool for detecting patients susceptible to adverse outcomes and complications (7). These vital signs serve as crucial indicators for triaging and signaling the necessity for emergency care, follow-up, and subsequent clinical decisions and play a pivotal role in the National Early Warning Score (NEWS/NEWS2) system (8). However, despite their widespread clinical utility, the predictive accuracy of an initial NEWS/NEWS2 score for in-hospital mortality in COVID-19 patients seem to be lower compared to non-COVID-19 patients. Moreover, the majority of studies investigating how vital signs, and the NEWS/NEWS2 score are associated with adverse events were conducted in the beginning of the COVID-19 pandemic (9–16). Furthermore, certain vital sign patterns may vary among different demographic groups, such as age, sex, and comorbidities, underscoring the importance of tailored risk assessment approaches (17, 18).

To advance our understanding of the role of physiological vital signs included in NEWS/NEWS2 scores in risk stratification of COVID-19 patients admitted to emergency care, studies incorporating comprehensive data spanning longer periods of the pandemic are needed. The aim of the present study was to investigate whether physiological vital signs measured at admission, including heart rate, respiratory rate, systolic blood pressure, saturation, and temperature, are associated with the risk of intermediate care unit (IMCU) and intensive care unit (ICU) admission and in-hospital mortality among patients with COVID-19 as their primary cause of hospital admission. Furthermore, we explored variations in these associations across the initial three waves of the COVID-19 pandemic to reflect the evolving landscape with the new SARS-CoV-2 variants as well as available vaccines and new clinical practices.

## Methods

### Study population

**Figure 1:**
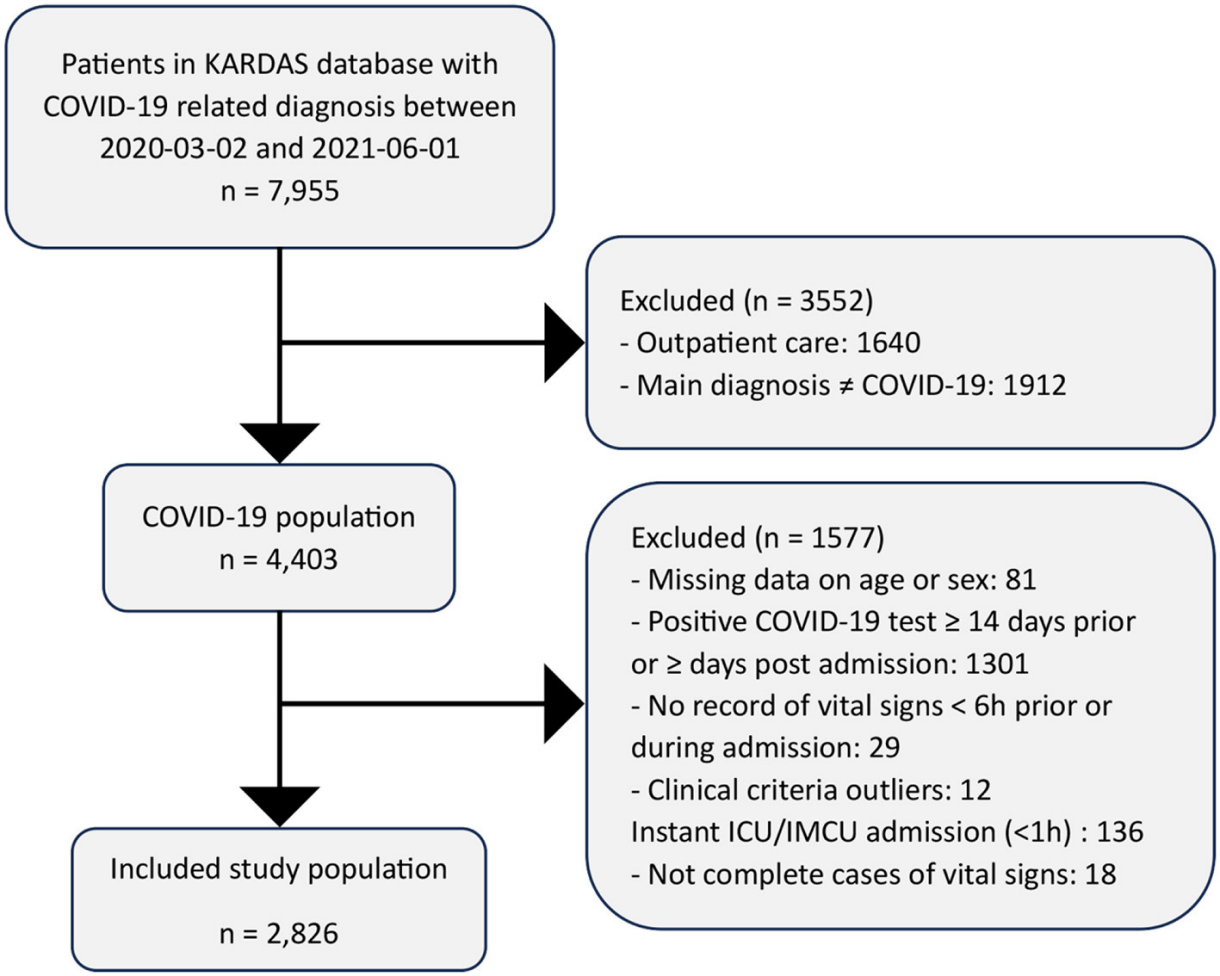
Inclusion and exclusion criteria for the study cohort.

This retrospective cohort study of patients admitted to Karolinska University Hospital (KUH), which has two main campuses located in northern and southern Stockholm, respectively, between 2 March 2020 and 1 June 2021. Figure 1 represents a flow chart of the included patients. Retrospective data were retrieved from the KARDAS database. The KARDAS database is an electronic health record database including summary electronic health records (EHR), therapeutic investigations and diagnosis during hospital stay. The study population included all adult patients ≥18 years who were admitted to the hospitals with COVID-19 as the primary diagnosis. Primary diagnosis of COVID-19 was defined as the presence of the ICD-10-SE U07.1 COVID-19, a virus identified in medical records and a positive PCR test for SARS-CoV-2, ranging from 14 days prior to admission up to 3 days after admission. Patients were excluded if they had missing information on vital signs, sex or age (*n* = 99). We further excluded patients admitted to the ICU or intermediate care unit (IMCU) within 1 h of admission (*n* = 136) to avoid influence of reversed causality. Thus, 2,826 patients were included in our final analysis.

Three time periods were categorized to cover separate waves of hospital admissions (wave 1: 2 March 2020–30 September 2020; wave 2: 1 October 2020–31 January 2021; and wave 3: 1 February−1 June 2021). The time periods for the first and second waves were defined to be consistent with those from the analysis by the Swedish National Board of Health and Welfare (19). The third wave was defined as the data remaining after 31 January 2021.

### Vital signs

In this study physiological vital signs included in the NEWS score were investigated to reflect early risk stratification during triaging taking place at the emergency ward. Vital signs at admission were defined as the first vital signs registered up to 6 h prior to admission including respiratory rate (breaths/min), heart rate (beats/min), systolic blood pressure (SBP, mmHg), temperature (°C) and oxygen saturation (SpO_2_) (%).

### Potential confounders

We collected data on age, sex, and medication use prior to admission, and the use of oxygen support (L/min). Age and sex were identified from the Swedish personal identification number. Medication use prior to admission was used as a proxy for other comorbidities at baseline. Medications were grouped on the Anatomical Therapeutic Chemical/Defined Daily Dose (ATC/DDD) classification, which is used by the World Health Organization (20). We included antihypertensive medication use, which was defined as the use of ACE inhibitors (ATC code C09AA), ARBs (C09C and C09D), or calcium channel blockers (C08). The anticoagulants used were warfarin (B01AA03), low-molecular-weight heparin (B01AB04, B01AB05, B01AB06, B01AB07, B01AB08, and B01AB10), or novel oral anticoagulants (B01AE07). High-cholesterol medications are defined as statins (C10AAs). Platelet inhibitors are defined as platelet aggregation inhibitors excluding heparin (B01AC). Medications were prescribed before hospital admission but could be extended during the hospital stay. For oxygen support we only had available information on volume of supplementary oxygen at arrival to the emergency department. We did not have available information on type of oxygen supplementation.

### Outcomes

Cases of ICU or IMCU admission and in-hospital death were identified from the KARDAS database. This contain detailed information on which units' patients were admitted to and time of referral to another using including ICU and IMCU including date and time. At Karolinska University Hospital, IMCU and ICU are defined as units that provide inotropic and non-invasive or invasive respiratory treatment. The time to event was calculated as the number of hours from admission to the ICU or IMCU, the time of death or discharge, or the end of the study period (1st of June 2021), whichever came first.

### Statistical analysis

Descriptive statistics are presented as median and interquartile range (IQR) for continuous variables and percentages (%) for categorical variables. Boxplots were constructed for graphical presentation of vital signs. Multicollinearity was explored by calculating Pearson correlation coefficients between vital signs. Cox proportional hazard models were used to calculate crude and multivariable-adjusted hazard ratios (HRs) with 95% confidence intervals (CIs) for the association between vital signs at admission and ICU/IMCU admission and in-hospital mortality. We designed two multivariable adjusted models. Model 1 was adjusted for age, sex, medications, supplementary oxygen flow (L/min). Model 2 was adjusted for age, sex, medications, supplementary oxygen flow (L/min) and all vital signs simultaneously. Selection of confounders was based on a priori knowledge and what was available from the medical records. Missing information on variables was handled using the complete case approach. In total, 81 participants had missing information on age and sex as they lacked a Swedish personal identification number and 18 did not have complete information on vital signs (Figure 1). Proportional hazard assumptions were evaluated with the Schoenfeld test and graphical examination of each variable. Violations in the proportional hazard assumption were observed for sex; therefore, all adjusted models were stratified for this variable. In the subgroup analysis, we investigated whether the association between vital signs at admission and ICU/IMCU admission and in-hospital mortality varied according to timing of admission (three time periods reflecting waves of the pandemic). To investigate whether the associations were modified by potential risk factors for severe COVID-19, we performed subgroup analyses by age (<65 or ≥65 years), gender (woman or man), antihypertensive medication (no or yes), anticoagulants (no or yes), lipid-lowering drugs (no or yes), and platelet inhibitors (no or yes), and oxygen support (no or yes). Multiplicative interactions were investigated with Wald chi-square tests. Analysis was performed in RStudio and R version 4.2.1 via the survival package.

## Results

**Figure 2:**
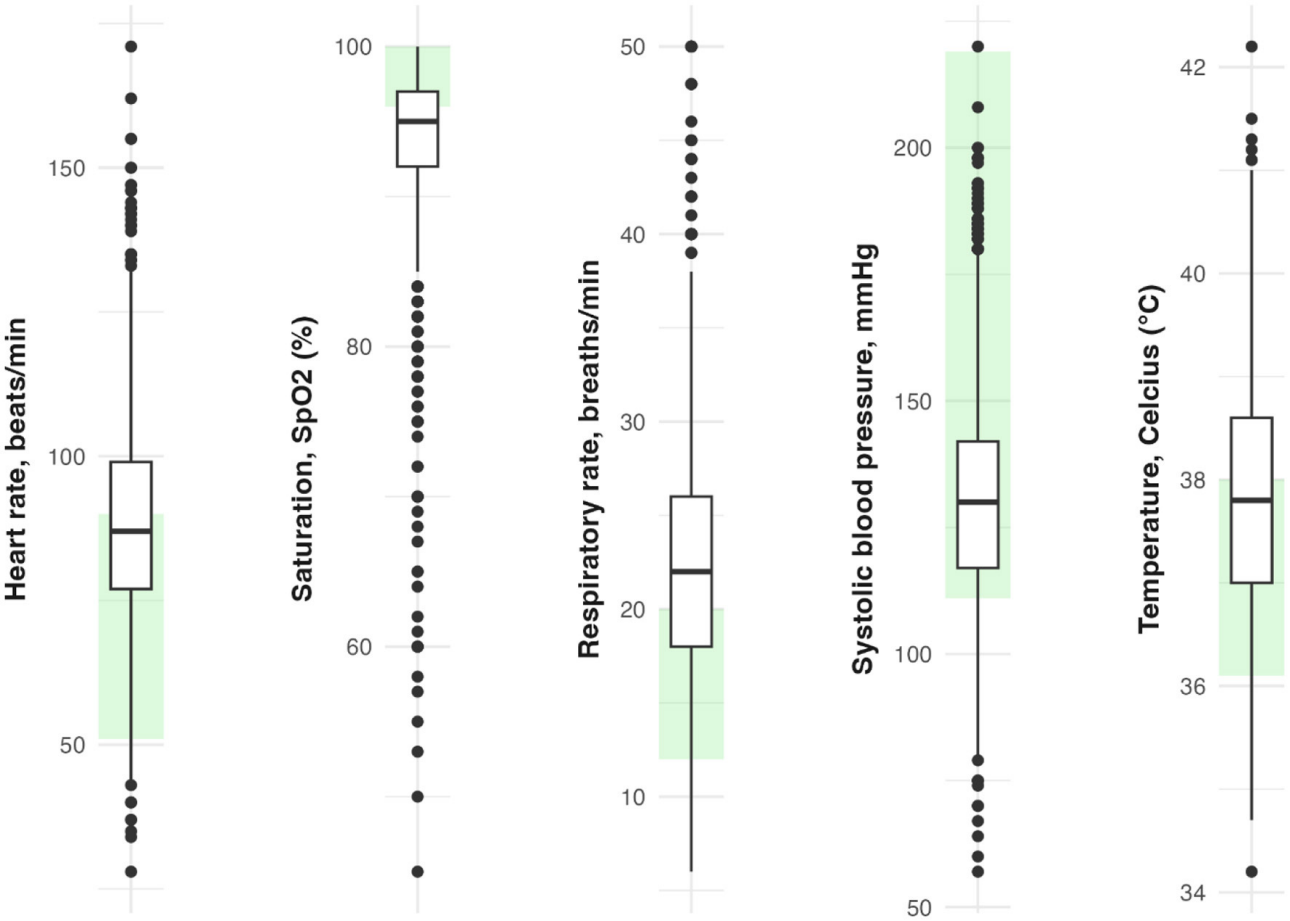
Distribution of vital signs at admission. Respiratory rate (breaths/min), systolic blood pressure (mmHg), heart rate (beats/min), temperature (°C) and oxygen saturation (%). A total of 214 (7.6%) patients had supplementary oxygen at the first measurement of saturation. The shaded area (light green) represents normal ranges according to National Early Warning Score 2 (NEWS2).

**Figure 3:**
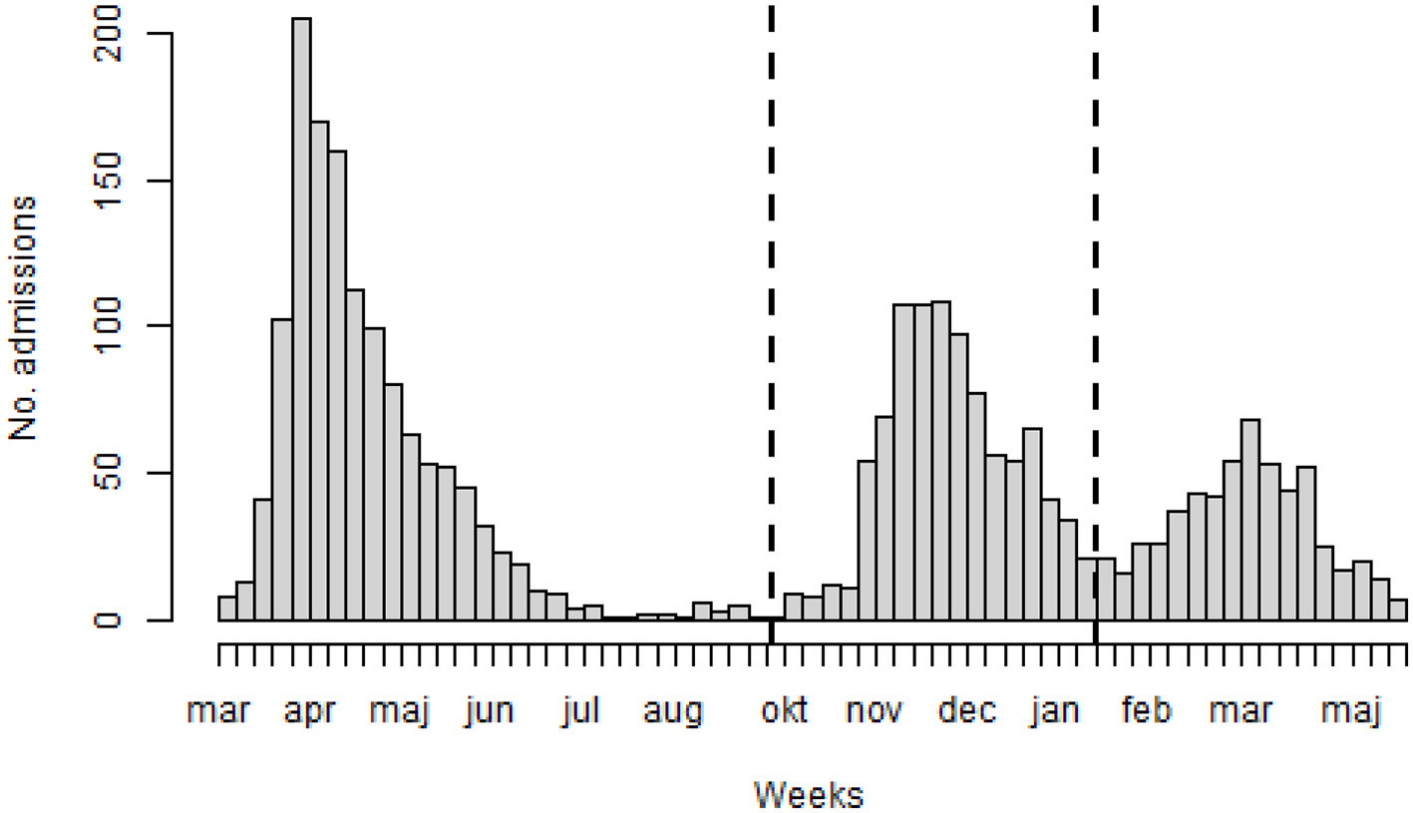
Number of admissions per week between 2 March 2020–1 June 2021. The dotted lines represent the starting dates of wave 2 (1 October 2020) and wave 3 (1 February 2021).

The characteristics of the study population are presented in Table 1. The median hospital stay was 6.98 days. The median age was 62.2 years (IQR 51–75), and 62.1% were men. During the follow-up, 246 (8.7%) patients were admitted to the IMCU, and 241 (8.5%) were admitted to the ICU. A total of 296 in-hospital deaths were recorded. At admission, the median respiratory rate was 22 breaths/min (IQR 18–26), the median body temperature was 37.8°C (IQR 37.0–38.6), the median heart rate was 87 beats/min (IQR 77–99), the median systolic blood pressure was 130 mmHg (IQR 117–142), and the median oxygen saturation was 95% (IQR 92–97; Figure 2). In Supplemental Figure 1, the Pearson correlation coefficient between vital signs is presented. The highest correlation coefficient was observed between respiratory rate and saturation (*r* = 0–29) followed by temperature and heart rate (*r* = 0.26). Figure 3 depicts weekly hospital admissions according to the three pandemic waves and starting dates for waves 2 and 3. There were 1,327 patients in the first wave, 931 in the second wave, and 568 in the third wave.

**Table 1:** Baseline characteristics of the study cohort.

**Time periods**	**Total (*N* = 2826)**	**Wave 1: 2020-03-02 to 2020-09-30 (*N* = 1327)**	**Wave 2: 2020-10-01 to 2021-01-31 (*N* = 931)**	**Wave 3: 2021-02-01 to 2021-06-01 (*N* = 568)**
**Age (years)**	62.2 [51–75]	60.5 [49–73]	65.6 [55–78]	60.4 [49–73]
**Gender**				
Women	1,070 (37.9%)	476 (35.9%)	381 (40.9%)	213 (37.5%)
Men	1,756 (62.1%)	851 (64.1%)	550 (59.1%)	355 (62.5%)
**Vital signs**				
Respiratory rate (breaths/min)	22.0 [18–26]	22.0 [18–26]	21.0 [18–25]	21.0 [18–25]
Systolic Blood Pressure (mmHg)	130 [117–142]	130 [116–141]	130 [118–145]	130 [116–142]
Heart Rate (bpm)	87.0 [77.0–99.0]	88.0 [78.0–100.0]	86.0 [75.0–97.0]	88.0 [78.0–98.0]
Temperature (°C)	37.8 [37.0–38.6]	37.9 [37.1–38.7]	37.6 [36.9–38.4]	37.7 [37.0–38.6]
Saturation (%)	95.0 [92.0–97.0]	95.0 [92.0–97.0]	95.0 [92.0–97.0]	95.0 [93.0–97.0]
Oxygen at admission	214 (7.6%)	83 (6.3%)	94 (10.1%)	37 (6.5%)
**Medications** ^a^				
Antihypertensives	515 (18.2%)	240 (18.1%)	176 (18.9%)	99 (17.5%)
Anticoagulants	422 (14.9%)	147 (11.1%)	175 (18.8%)	100 (17.6%)
High cholesterol	241 (8.5%)	111 (8.4%)	85 (9.1%)	45 (8.0%)
Platelet inhibitors	213 (7.5%)	102 (7.7%)	82 (8.8%)	29 (5.1%)
In–hospital deaths	296 (10.5%)	174 (13.1%)	83 (8.9%)	39 (6.9%)
**Critical care**				
ICU admission	241 (8.5%)	135 (10.2%)	70 (7.5%)	36 (6.3%)
IMCU admission	246 (8.7%)	98 (7.4%)	80 (8.6%)	68 (12.0%)
**Length of stay**				
Hospital (days)	6.98 [3.55–12.60]	6.65 [3.50–11.90]	7.55 [3.57–12.90]	7.35 [3.64–13.10]
ICU (days)	8.12 [3.79–16.9]	9.73 [4.35–17.30]	7.77 [3.41–18.20]	5.21 [3.81–10.80]
IMCU (days)	2.91 [1.42–4.99]	2.49 [1.23–4.16]	3.11 [1.69–5.23]	2.97 [1.51–5.94]
**Time–to–event**				
Discharge (days)	5.86 [2.85–9.71]	5.60 [2.84–9.27]	6.29 [2.94–10.30]	5.91 [2.76–9.73]
Death (days)	7.00 [4.33–12.70]	6.23 [4.17, 9.12]	8.39 [4.41–15.50]	15.6 [11.5–18.30]
ICU or IMCU admission (days)	1.75 [0.569–3.94]	1.31 [0.493–3.17]	2.45 [0.814–4.58]	1.79 [0.499– 4.15]

Continuous variables are presented as medians (IQRs). Categorical variables are presented as numbers (percentages).

a Medication use at admission. Antihypertensive medication use was defined as the use of ACE inhibitors, ARBs, or calcium channel blockers. Anticoagulants: Warfarin, low-molecular-weight heparin, or novel oral anticoagulants. High-cholesterol medications: statins. Platelet inhibitors: Platelet aggregation inhibitors excluding heparin.

### Associations between vital signs at admission and the risk of ICU/IMCU admission

Table 2 shows the HRs and 95% CIs for ICU or IMCU admission. In the crude analysis, respiratory rate (HR 1.06, CI 1.05–1.07), heart rate (HR 1.01, CI 1.01–1.02), temperature (HR 1.29, CI 1.19–1.40), and lower oxygen saturation (HR 1.06, CI 1.05–1.07) were associated with a higher risk of ICU/IMCU admission. Adjustments for age, sex, medication use, and oxygen (L/min) showed similar associations. When all vital signs were adjusted for simultaneously, respiratory rate (HR 1.03, CI 1.02–1.05), temperature (HR 1.21 CI 1.11–1.32) and lower oxygen saturation (HR 1.05 CI 1.04–1.06) were associated with a higher risk of ICU/IMCU admission. Systolic blood pressure was not associated with the ICU/IMCU in any model. In sensitivity analysis, we included patient that were directly transferred to ICU/IMCU (*N* = 136) that were excluded in the main analysis, and the results remained similar except for temperature [HR: 1.13 (95% CI: 1.04–1.06)] that was slightly attenuated. In the subgroup analysis of various pandemic waves, similar associations were observed; however, only respiratory rate and saturation remained statistically significant across all three waves. In Supplementary Table 1, the association between vital signs and ICU/IMCU admission by subgroups of age, gender, baseline medication use, and oxygen supply are shown. Statistically significant interaction effects were observed saturation and age (*P* for interaction = 0.000), heart rate and age (*P* for interaction = 0.01), temperature and anticoagulants (*P* for interaction = 0.01) total mortality (*P* = 0.04 for interaction).

**Table 2:** Hazard ratios for the association between each unit change in vital signs at admission and ICU or IMCU admission.

**95% confidence interval**	**Events**	**Crude**	**Multivariable-adjusted model 1^a^**	**Multivariable-adjusted model 2^b^**
**Vital signs (*****N*** = **2,826)**				
Respiratory rate (breaths/min)	487	1.06 (1.05–1.07)	1.06 (1.04–1.07)	1.03 (1.02–1.05)
Saturation (%)	487	1.06 (1.05–1.07)	1.06 (1.05–1.07)	1.05 (1.04–1.06)
Systolic blood pressure (mmHg)	487	1.00 (0.99–1.00)	1.00 (0.99–1.00)	1.00 (0.99–1.00)
Heart rate (bpm)	487	1.01 (1.01–1.02)	1.01 (1.01–1.02)	1.00 (1.00–1.01)
Temperature (°C)	487	1.29 (1.19–1.40)	1.29 (1.18–1.39)	1.21 (1.11–1.32)
**Vital signs, wave 1 (*****N*** = **1,327)**				
Respiratory rate (breaths/min)	233	1.06 (1.04–1.08)	1.05 (1.03–1.07)	1.02 (1.00–1.05)
Saturation (%)	233	1.06 (1.04–1.07)	1.06 (1.05–1.08)	1.06 (1.04–1.07)
Systolic blood pressure (mmHg)	233	1.00 (0.99–1.00)	1.00 (0.99–1.01)	1.00 (0.99–1.00)
Heart rate (bpm)	233	1.01 (1.00–1.02)	1.01 (1.00–1.02)	1.00 (0.99–1.01)
Temperature (°C)	233	1.35 (1.21–1.52)	1.34 (1.19–1.50)	1.29 (1.14–1.46)
**Vital signs, wave 2 (*****N*** = **931)**				
Respiratory rate (breaths/min)	150	1.06 (1.04–1.08)	1.05 (1.03–1.08)	1.03 (1.01–1.06)
Saturation (%)	150	1.05 (1.03–1.07)	1.05 (1.03–1.07)	1.04 (1.01–1.06)
Systolic blood pressure (mmHg)	150	1.00 (0.99–1.00)	1.00 (0.99–1.00)	1.00 (0.99–1.00)
Heart rate (bpm)	150	1.01 (1.00–1.02)	1.01 (1.00–1.02)	1.01 (1.00–1.02)
Temperature (°C)	150	1.25 (1.08–1.45)	1.26 (1.09–1.47)	1.17 (1.00–1.37)
**Vital signs, wave 3 (*****N*** = **565)**				
Respiratory rate (breaths/min)	106	1.06 (1.03–1.09)	1.07 (1.04–1.10)	1.05 (1.02–1.08)
Saturation (%)	106	1.08 (1.05–1.10)	1.08 (1.05–1.11)	1.06 (1.03–1.09)
Systolic blood pressure (mmHg)	106	1.00 (0.99–1.01)	1.00 (0.99–1.01)	1.00 (0.99–1.01)
Heart rate (bpm)	106	1.01 (1.00–1.02)	1.01 (1.00–1.02)	1.00 (0.99–1.02)
Temperature (°C)	106	1.18 (0.98–1.42)	1.21 (1.00–1.46)	1.14 (0.94–1.38)

a Multivariate adjusted Model 1 adjusted for age, sex (stratified), medications (antihypertensives, anticoagulants, lipid-lowering drugs, and platelet inhibitors), and oxygen support (L/min) at the first vital sign measurement.

b Multivariable-adjusted Model 2 adjusted for age, sex (stratified), medications (antihypertensives, anticoagulants, lipid-lowering drugs, and platelet inhibitors), and oxygen support (L/min) at the first vital sign measurement and all vital signs simultaneously.

Note that in Table 1, we included all patients admitted to the ICU or IMCU during the entire observation period. However, in the model, given that ICU admission occurs after the IMCU, it will appear as one event. Hence, the number of events is smaller in Table 2 than in Table 1.

### Associations between vital signs, admission and in-hospital mortality

The associations between vital signs and in-hospital mortality are shown in Table 3. In the crude analysis, a higher (every unit change) respiratory rate (breaths/min; HR 1.07, 95% CI 1.05–1.09) and lower saturation (HR 1.07, 95% CI 1.05–1.09) were associated with higher in-hospital mortality. Further adjustment for age, sex, medications and oxygen (L/min) showed similar associations. Additional adjustments for vital signs slightly attenuated the associations for respiratory rate (HR 1.04, 95% CI: 1.02–1.07) and saturation (HR 1.04, 95% CI: 1.02–1.06). Systolic blood pressure, heart rate, temperature and oxygen saturation were not significantly associated with in-hospital mortality. In sensitivity analysis, patient that were directly transferred to ICU/IMCU were included and similar the results were observed except for temperature [HR: 1.02 (95% CI: 1.00–1.03)]. In the analysis across the waves, statistically significant associations were observed for lower saturation across all three waves whereas the association with respiratory rate was statistically significantly associated with higher in-hospital mortality in wave 1. There was statistically significant interaction effects observed between saturation and use of platelet inhibitors at baseline (*P* for interaction = 0.04), and saturation and oxygen support (*P* for interaction = 0.03; Supplementary Table 2).

**Table 3:** Hazard ratios (95% confidence intervals) for the associations between every unit change in vital signs at admission and in-hospital mortality.

**95% confidence interval**	***N***	**Crude**	**Multivariable-adjusted model 1^a^**	**Multivariable-adjusted model 2^b^**
**Vital signs (events: 296)**				
Respiratory rate (breaths/min)	2,826	1.07 (1.05–1.09)	1.07 (1.05–1.09)	1.04 (1.02–1.07)
Saturation (%)	2,826	1.07 (1.05–1.09)	1.06 (1.04–1.08)	1.04 (1.02–1.06)
Systolic blood pressure (mmHg)	2,826	1.00 (0.99–1.00)	0.99 (0.98–1.00)	0.99 (0.98–1.00)
Heart rate (bpm)	2,826	1.00 (0.99–1.01)	1.01 (1.00–1.01)	1.00 (0.99–1.01)
Temperature (°C)	2,826	1.06 (0.92–1.22)	1.19 (1.03–1.38)	1.12 (0.96–1.30)
**Vital signs, wave 1 (events: 174)**				
Respiratory rate (breaths/min)	1,327	1.07 (1.05–1.10)	1.07 (1.05–1.10)	1.05 (1.02–1.09)
Saturation (%)	1,327	1.08 (1.05–1.10)	1.05 (1.03–1.08)	1.04 (1.01–1.07)
Systolic blood pressure (mmHg)	1327	1.00 (0.99–1.00)	0.99 (0.98–1.00)	0.99 (0.99–1.00)
Heart rate (bpm)	1,327	1.00 (0.99–1.01)	1.00 (0.99–1.01)	0.99 (0.98–1.00)
Temperature (°C)	1,327	1.06 (0.89–1.26)	1.21 (1.01–1.44)	1.13 (0.93–1.38)
**Vital signs, wave 2 (events: 83)**				
Respiratory rate (breaths/min)	931	1.05 (1.00–1.10)	1.04 (0.99–1.09)	1.01 (0.95–1.06)
Saturation (%)	931	1.05 (1.02–1.09)	1.06 (1.02–1.10)	1.06 (1.02–1.10)
Systolic blood pressure (mmHg)	931	1.00 (0.99–1.02)	1.00 (0.98–1.01)	1.00 (0.98–1.01)
Heart rate (bpm)	931	1.00 (0.98–1.02)	1.00 (0.99–1.02)	1.00 (0.99–1.02)
Temperature (°C)	931	1.10 (0.83–1.46)	1.22 (0.89–1.65)	1.14 (0.83–1.57)
**Vital signs, wave 3 (events: 39)**				
Respiratory rate (breaths/min)	568	1.06 (0.97–1.15)	1.08 (0.99–1.17)	1.00 (0.92–1.08)
Saturation (%)	568	1.13 (1.07–1.20)	1.15 (1.07–1.23)	1.15 (1.08–1.22)
Systolic blood pressure (mmHg)	568	0.99 (0.97–1.01)	0.99 (0.97–1.02)	0.99 (0.97–1.01)
Heart rate (bpm)	568	0.99 (0.96–1.02)	1.01 (0.98–1.04)	1.01 (0.98–1.03)
Temperature (°C)	568	0.66 (0.40–1.10)	1.26 (0.71–2.24)	1.36 (0.91–2.04)

a Multivariate adjusted Model 1 adjusted for age, sex (stratified), medications (antihypertensives, anticoagulants, lipid-lowering drugs, and platelet inhibitors), and oxygen support (L/min) at the first vital sign measurement.

b Multivariable-adjusted Model 2 adjusted for age, sex (stratified), medications (antihypertensives, anticoagulants, lipid-lowering drugs, and platelet inhibitors), and oxygen support (L/min) at the first vital sign measurement and all vital signs simultaneously.

## Discussion

In this retrospective cohort study of COVID-19 patients admitted to two Karolinska University Hospitals, several vital signs at admission were associated with ICU/IMCU admission and in-hospital mortality. Among all vital signs investigated, a higher respiratory rate and lower saturation were associated with both admission to the ICU/IMCU and in-hospital mortality. In addition, increased temperature and heart rate were associated with an increased risk of admission to the ICU/IMCU. The associations were similar across all the pandemic waves.

The present study adds to the accumulating evidence on the use of vital signs at admission for the triage of COVID-19 patients. Close monitoring of vital signs is important for identifying patients at risk of adverse events, including ICU/IMCU admission and in-hospital mortality. Although adverse events among COVID-19 patients have decreased with the development of treatments and vaccines (21), patients are still admitted to hospitals and ICU/IMCU due to COVID-19.

To the best of our knowledge no previous study have investigated the association between vital signs at admission and risk of higher-level care including ICU/IMCU admission. However, one cross-sectional study investigated the association between NEWS2 and severe and critical illness state among 414 adults admitted to hospital care in China (22). In this study, NEWS2 score >2 was associated with higher odds of being in a severe/critical state. Consistent with our findings, patients considered severe and critical ill had lower saturation, higher respiratory rate, and higher temperature. In another cohort study conducted in Norway, NEWS2 score showed and area-under-the-curve of 0.822 for severe COVID-19 among 66 patients admitted a general hospital. Also, in this study lower saturation and higher respiratory rate was associated with the severity of COVID-19 (23). Thus, our study, in a bigger patient cohort, confirm and extend the importance of vital scores at admission to predict severe disease, need for therapeutic support and risk for adverse outcome.

More studies has been conducted on the association between vital signs and/or NEWS2 scores and in-hospital mortality (11, 14–16, 24). The majority of these studies were conducted at the beginning of the pandemic and have not taking the evolving landscape with regard to introduction of vaccines and new treatments into account. Our findings are consistent with another Swedish cohort study conducted early in the pandemic among 1,785 geriatric patients reporting that lower saturation (<90%), but not temperature, systolic and diastolic blood pressure or pulse, was associated with in-hospital death (16). Our findings are also in line with three previous studies conducted in the US reporting that higher heart rate, higher respiratory rate, and lower oxygen saturation were related to COVID-19 or in-hospital mortality (14, 15, 24). In addition, one previous study of 1393 COVID-19 patients admitted to six Apollo hospitals in India observed that respiratory rate (>24/min) and oxygen level below 90% (collected at or within 24h following admission) were associated with higher in-hospital mortality (11). This is in line with an early (June 2020) retrospective cohort study of 140 patients in Wuhan exhibiting the association of hypoxemia (oxygen level below 90%) and in-hospital mortality (25). Thus, the present data that the association between increased respiratory rate and lower saturation at admittance (<6 h) for COVID-19 confirm and extend the results from these earlier studies. Furthermore, we present novel data regarding the association between respiratory rate and saturation at admission for COVID-19, that remain across the pandemic waves and hence different SARS-CoV-2 variants.

Higher temperature was associated with both IMCU/ICU admission and in-hospital mortality; however, the association with in-hospital mortality was not significant when other vital signs were further adjusted for. It is known that temperature is correlated with both the respiratory rate and heart rate (26). Our observations are in line with those of a cohort study including 7,614 COVID-19 patients from New York hospitals (27). Here higher body temperature at the initial presentation did not show a significant association to mortality. Notably in this study patients presenting with body temperature ≤ 36 °Celsius had the highest mortality. In this in-hospital study and another study of 102 mechanically ventilated patients in the US, increasing peak temperature (>103 Fahrenheit, circa 39.44 Celsius), during hospital stay, was associated with mortality (28).

We did not observe any association between SBP and in-hospital mortality. This is in contrast to a study of 157 patients in Wuhan, China, that reported that a higher SBP at admission was a predictor of mortality (29). In addition, a Spanish study of more than 12,000 patients observed that hypertension among patients treated with angiotensin-converting enzyme inhibitors or renin-angiotensin-aldosterone blockers or angiotensin II receptor blockers was significantly associated with lower all-cause mortality (30).

The present results align with and extend initial studies of COVID-19 patients. Thus, respiratory variables including oxygen saturation and respiratory rate are associated with severity of COVID-19 and mortality (31). They may therefore be used to evaluate and potentially refine current early warning scores, such as the NEWS/NEWS2 and the Sequential Organ Failure Assessment (SOFA) score. This can improve risk stratification during triaging and care of COVID-19 and likely other patients with potential life-threatening viral sepsis. Clinically, this highlights the utility of close monitoring and rapid response to changes in respiratory rate and oxygen saturation at the time of admission. It is well-documented that ARDS was a major complication among COVID-19 patients. However, the clinical symptoms of COVID-19-related respiratory distress were perceived to differ from those caused by other well-known pathogens and non-COVID-19 ARDS (32). Early observations in the pandemic indicated that patients were admitted to emergency departments with minimal or no respiratory distress but found to have severe hypoxia upon further examination (33, 34). This phenomenon indicates that severe oxygen deprivation can occur without typical signs of respiratory distress, revealing a critical aspect of the clinical presentation of viral sepsis, including COVID-19. This is referred to as silent hypoxia, or hidden hypoxemia, where patients present. Notably, silent hypoxia may not only be an identifiable physiological phenotype of COVID-19 ARDS, but is also observed among patients with other causes of respiratory failure (35). Furthermore, dyspnoea is not related to hypoxemia, but is more closely related to inspiratory drive and mechanical alterations (34).

The mechanisms behind silent hypoxemia in COVID-19 and other patients with respiratory falure involve complex physiological responses. These include a combination of impaired peripheral and central oxygen sensing (34). Moreover, hypoxia without respiratory distress typical of ARDS, in some COVID-19 patients might be due to preserved lung compliance (36). Importantly, the lack of dyspnea in the early stages of the disease is likely related to the absence of increased inspiratory drive due to compensatory mechanisms of hypoxemia. Thus, adequately measuring the respiratory rate, increased during hypoxia, is an essential and early indicator of disease and potential adverse outcome. Notably, respiratory rate increase in response to inflammation, viral sepsis and pneumonia before saturation decrease. Therefore, the increased respiratory rate observed in these patients may serve as an early indicator of deteriorating respiratory function. This sensitivity of respiratory frequency, in detecting subtle decreases in oxygen levels, highlights the importance of vigilant monitoring of vital signs, as traditional symptoms may not always reflect the severity of hypoxia (33). Recognizing these mechanisms is crucial for improving early warning systems and implementing timely interventions for at-risk patients.

Although several factors changed during the pandemic course, including policies (37), therapeutic strategies (38), virus variants and vaccines (39), we still observed similar associations for ICU/IMCU admission and in-hospital mortality across the pandemic waves. However, the background characteristics slightly changed according to age and comorbidities. In addition, the associations between saturation, heart rate and ICU/IMCU admission varied according to age and where somewhat stronger associations were observed among patients <65 years. This may be due to that other comorbidities and medication use may impact the association between vital signs and risk of ICU/IMCU admission since these factors are more common among patients with older age. Moreover, we also observed a statistically significant interaction effect between saturation and oxygen support for in-hospital mortality. Where a stronger association for every unit decrease in saturation and increased in-hospital mortality was observed among patients already receiving oxygen support. This may be explained by that patients on oxygen support and still desaturating may reflect a more severe COVID-19.

Future research should focus on understanding the dynamic changes in vital signs throughout hospital stays, which could further enhance predictive models and aid clinicians in identifying patients at risk of deterioration earlier in their clinical course. Such efforts could lead to more tailored and timely interventions, ultimately improving patient outcomes in the evolving landscape of COVID-19 management.

The strengths of this study include its large sample size, extended time with several pandemic waves, and detailed data retrieved from medical records. Our study also has several limitations. We cannot exclude the possibility that uncontrolled and residual confounding factors may have had an impact on our results. We had limited information on comorbidities before admission. Thus, medications were used as proxies for comorbidities. We also had missing information on BMI at admission for many participants and did not include this variable in our analysis. In addition, we cannot exclude that vaccine status could impact the associations between vital signs, ICU/IMCU admission and in-hospital mortality. However, this information was not available and instead subgroup analyses of the pandemic waves were performed to reflect the introduction of vaccines and new treatment strategies. Other covariates that may be important include ethnicity and demographics. The study population included patients from two Karolinska University Hospital sites, which may limit the generalizability of our results. However, as similar association have been observed in other settings including US (14, 15, 24), India (11), and China (25) we believe external validity is a minor issue. In addition, the measurement of physiological vital signs is a simple low-cost procedure and may be easily adapted to low-resource settings. The results from this study may not represent later pandemic viral variants (40) or new therapies (38).

In conclusion, this study suggests that respiratory-related vital signs at admission, especially respiratory rate and saturation are associated with an increased risk of both ICU/IMCU admission and in-hospital mortality. These results may support more emphasis on respiratory rate and saturation in early warning scores, such as the NEWS/NEWS2 and SOFA score. Future studies of longitudinal changes in vital signs could aid in identifying COVID-19 patients at risk of clinical deterioration.

## Data Availability

The data analyzed in this study is subject to the following licenses/restrictions: Since the datasets are personal health records (PHRs). Requests to access these datasets should be directed to eric.herlenius@ki.se.

## References

[1] Lamers MM Haagmans BL. SARS-CoV-2 pathogenesis. Nat Rev Microbiol. (2022) 20:270–84. 10.1038/s41579-022-00713-0 35354968

[2] Update to living systematic review on prediction models for diagnosis and prognosis of covid-19. BMJ. (2022) 378:o2009. 10.1136/bmj.o2009 35995453

[3] Tang N Li D Wang X Sun Z. Abnormal coagulation parameters are associated with poor prognosis in patients with novel coronavirus pneumonia. J Thromb Haemost. (2020) 18:844–7. 10.1111/jth.14768 32073213 PMC7166509

[4] Lu Y Ao D He X Wei X. The rising SARS-CoV-2 JN.1 variant: evolution, infectivity, immune escape, and response strategies. MedComm. (2024) 5:e675. 10.1002/mco2.675 39081516 PMC11286544

[5] Planas D Veyer D Baidaliuk A Staropoli I Guivel-Benhassine F Rajah MM. Reduced sensitivity of SARS-CoV-2 variant Delta to antibody neutralization. Nature. (2021) 596:276–80. 10.1038/s41586-021-03777-9 34237773

[6] Viana R Moyo S Amoako DG Tegally H Scheepers C Althaus CL. Rapid epidemic expansion of the SARS-CoV-2 Omicron variant in Southern Africa. Nature. (2022) 603:679–86. 35042229 10.1038/s41586-022-04411-y PMC8942855

[7] Brekke IJ Puntervoll LH Pedersen PB Kellett J Brabrand M. The value of vital sign trends in predicting and monitoring clinical deterioration: A systematic review. PLoS ONE. (2019) 14:e0210875. 10.1371/journal.pone.0210875 30645637 PMC6333367

[8] Williams B. The National Early Warning Score: from concept to NHS implementation. Clin Med. (2022) 22:499–505. 10.7861/clinmed.2022-news-concept 36427887 PMC9761416

[9] Smith GB Prytherch DR Meredith P Schmidt PE Featherstone PI. The ability of the National Early Warning Score (NEWS) to discriminate patients at risk of early cardiac arrest, unanticipated intensive care unit admission, and death. Resuscitation. (2013) 84:465–70. 10.1016/j.resuscitation.2012.12.016 23295778

[10] Gong MN Bajwa EK Thompson BT Christiani DC. Body mass index is associated with the development of acute respiratory distress syndrome. Thorax. (2010) 65:44–50. 10.1136/thx.2009.117572 19770169 PMC3090260

[11] Kar S Chawla R Haranath SP Ramasubban S Ramakrishnan N Vaishya R. Multivariable mortality risk prediction using machine learning for COVID-19 patients at admission (AICOVID). Sci Rep. (2021) 11:12801. 10.1038/s41598-021-92146-7 34140592 PMC8211710

[12] Scott LJ Tavare A Hill EM Jordan L Juniper M Srivastava S. Prognostic value of National Early Warning Scores (NEWS2) and component physiology in hospitalised patients with COVID-19: a multicentre study. Emerg Med J. (2022) 39:589–94. 10.1136/emermed-2020-210624 35292484 PMC8931800

[13] Richardson D Faisal M Fiori M Beatson K Mohammed M. Use of the first National Early Warning Score recorded within 24 hours of admission to estimate the risk of in-hospital mortality in unplanned COVID-19 patients: a retrospective cohort study. BMJ Open. (2021) 11:e043721. 10.1136/bmjopen-2020-043721 33619194 PMC7902318

[14] Rechtman E Curtin P Navarro E Nirenberg S Horton MK. Vital signs assessed in initial clinical encounters predict COVID-19 mortality in an NYC hospital system. Sci Rep. (2020) 10:21545. 10.1038/s41598-020-78392-1 33298991 PMC7726000

[15] Sands KE Wenzel RP McLean LE Korwek KM Roach JD Miller KM. Patient characteristics and admitting vital signs associated with coronavirus disease 2019 (COVID-19)-related mortality among patients admitted with noncritical illness. Infect Control Hosp Epidemiol. (2021) 42:399–405. 10.1017/ice.2020.461 32928319 PMC7520636

[16] Xu H Garcia-Ptacek S Annetorp M Cederholm T Engel G Engstrom M. Decreased mortality over time during the first wave in patients with COVID-19 in geriatric care: data from the Stockholm GeroCovid Study. J Am Med Dir Assoc. (2021) 22:1565–73.e4. 10.1016/j.jamda.2021.06.005 34216553 PMC8196313

[17] Magesh S John D Li WT Li Y Mattingly-app A Jain S. Disparities in COVID-19 outcomes by race, ethnicity, and socioeconomic status: a systematic review and meta-analysis. JAMA Netw Open. (2021) 4:e2134147. 10.1001/jamanetworkopen.2021.34147 34762110 PMC8586903

[18] Chen N Zhou M Dong X Qu J Gong F Han Y. Epidemiological and clinical characteristics of 99 cases of 2019 novel coronavirus pneumonia in Wuhan, China: a descriptive study. Lancet. (2020) 395:507–13. 10.1016/S0140-6736(20)30211-7 32007143 PMC7135076

[19] Analys av första och andra covid-19-vågen –produktion köer och väntetider i vården Article Number 2021-5-7371 May 2021 Available online at: https://www.socialstyrelsen.se/publikationer/analys-av-forsta-och-andra-covid-19-vagen–produktion-koer-och-vantetider-i-varden-2021-5-7371/ (Accessed March 20, 2025).

[20] ATC/DDD Index 2022 Available online at: https://atcddd.fhi.no/atc_ddd_index/Oslo, Norway: WHO Collaborating Centre for Drug Statistics Methodology (2022). Available online at: https://atcddd.fhi.no/atc_ddd_index/ (Accessed March 20, 2025).

[21] Zhang G Tang T Chen Y Huang X Liang T. mRNA vaccines in disease prevention and treatment. Signal Transduct Target Ther. (2023) 8:365. 10.1038/s41392-023-01579-1 37726283 PMC10509165

[22] Li C Wang K Wu L Song B Tan J Su H. Prehospital physiological parameters related illness severity scores can accurately discriminate the severe/critical state in adult patients with COVID-19. Ann Med. (2023) 55:2239829. 10.1080/07853890.2023.2239829 37489620 PMC10392258

[23] Myrstad M Ihle-Hansen H Tveita AA Andersen EL Nygard S Tveit A. National Early Warning Score 2 (NEWS2) on admission predicts severe disease and in-hospital mortality from Covid-19 - a prospective cohort study. Scand J Trauma Resusc Emerg Med. (2020) 28:66. 10.1186/s13049-020-00764-3 32660623 PMC7356106

[24] Chatterjee NA Jensen PN Harris AW Nguyen DD Huang HD Cheng RK. Admission respiratory status predicts mortality in COVID-19. Influenza Other Respi Viruses. (2021) 15:569–72. 10.1111/irv.12869 34028169 PMC8242415

[25] Xie J Covassin N Fan Z Singh P Gao W Li G. Association between hypoxemia and mortality in patients With COVID-19. Mayo Clinic proceedings. (2020) 95:1138–47. 10.1016/j.mayocp.2020.04.006 32376101 PMC7151468

[26] Heal C Harvey A Brown S Rowland AG Roland D. The association between temperature, heart rate, and respiratory rate in children aged under 16 years attending urgent and emergency care settings. Eur J Emerg Med. (2022) 29:413–6. 10.1097/MEJ.0000000000000951 35679531 PMC9605188

[27] Tharakan S Nomoto K Miyashita S Ishikawa K. Body temperature correlates with mortality in COVID-19 patients. Critical Care. (2020) 24:298. 10.1186/s13054-020-03045-8 32503659 PMC7274509

[28] Choron RL Butts CA Bargoud C Krumrei NJ Teichman AL Schroeder ME. Fever in the ICU: a predictor of mortality in mechanically ventilated COVID-19 patients. J Intensive Care Med. (2021) 36:484–93. 10.1177/0885066620979622 33317374 PMC7738811

[29] Caillon A Zhao K Klein KO Greenwood CMT Lu Z Paradis P. High systolic blood pressure at hospital admission is an important risk factor in models predicting outcome of COVID-19 patients. Am J Hypertens. (2021) 34:282–90. 10.1093/ajh/hpaa225 33386395 PMC7799245

[30] Rodilla E Saura A Jimenez I Mendizabal A Pineda-Cantero A Lorenzo-Hernandez E. Association of hypertension with all-cause mortality among hospitalized patients with COVID-19. J Clin Med. (2020) 9:3136. 10.3390/jcm9103136 32998337 PMC7650567

[31] Kashani KB. Hypoxia in COVID-19: sign of severity or cause for poor outcomes. Mayo Clinic Proc. (2020) 95:1094–6. 10.1016/j.mayocp.2020.04.021 32498766 PMC7177114

[32] Matsumoto K Prowle JR Puthucheary Z Cecconi M Fazzini B Malcolm H. Uncertainty and decision-making in critical care: lessons from managing COVID-19 ARDS in preparation for the next pandemic. BMJ Open Respir Res. (2025) 12:e002637. 10.1136/bmjresp-2024-002637 40350182 PMC12067812

[33] Guo L Jin Z Gan TJ Wang E. Silent hypoxemia in patients with COVID-19 pneumonia: a review. Med Sci Monit. (2021) 27:e930776. 10.12659/MSM.930776 34635632 PMC8518510

[34] Simonson TS Baker TL Banzett RB Bishop T Dempsey JA Feldman JL. Silent hypoxaemia in COVID-19 patients. J Physiol. (2021) 599:1057–65. 10.1113/JP280769 33347610 PMC7902403

[35] Brusse-Keizer M Citgez E Zuur-Telgen M Kerstjens HAM Rijkers G VanderValk P. Difference in survival between COPD patients with an impaired immune reaction vs. an adequate immune reaction to seasonal influenza vaccination: the COMIC study. Respir Med. (2022) 197:106851. 10.1016/j.rmed.2022.106851 35487112

[36] Gattinoni L Coppola S Cressoni M Busana M Rossi S Chiumello D. COVID-19 does not lead to a “Typical” acute respiratory distress syndrome. Am J Respir Crit Care Med. (2020) 201:1299–300. 10.1164/rccm.202003-0817LE 32228035 PMC7233352

[37] Ludvigsson JF. How Sweden approached the COVID-19 pandemic: summary and commentary on the National Commission Inquiry. Acta Paediatr. (2023) 112:19–33. 10.1111/apa.16535 36065136 PMC9538368

[38] Roche N Crichton ML Goeminne PC Cao B Humbert M Shteinberg M. Update June 2022: management of hospitalised adults with coronavirus disease 2019 (COVID-19): a European Respiratory Society living guideline. Eur Respir J. (2022) 60:2200803. 10.1183/13993003.00803-2022 35710264 PMC9363848

[39] Nordstrom P Ballin M Nordstrom A. Risk of infection, hospitalisation, and death up to 9 months after a second dose of COVID-19 vaccine: a retrospective, total population cohort study in Sweden. Lancet. (2022) 399:814–23. 10.1016/S0140-6736(22)00089-7 35131043 PMC8816388

[40] Folkhälsomyndigheten. Varianter av viruset som orsakar covid-19. Available online at: https://www.folkhalsomyndigheten.se/smittskydd-beredskap/smittsamma-sjukdomar/covid-19/varianter-av-viruset-som-orsakar-covid-19 (Accessed March 20, 2025).

