# Supplementary material for: The association between vital signs at hospital admission and adverse outcomes in patients with COVID-19: a retrospective cohort study
Source: Front Med (Lausanne). 2025 Jul 3;12:1602129. doi: 10.3389/fmed.2025.1602129 (PMC12267188; doi:10.3389/fmed.2025.1602129)
Supplement: Supplementary file 1 [file Supplementary_file_1.docx]

**Supplemental Table 1:** **Vital signs and the risk of admission to ICU or IMCU by subgroups of potential risk factors of severe COVID-19**

|  | **Number of Participants** | **Number of events** | **Respiratory rate (breaths/min)** | **Saturation (%)** | **Systolic blood pressure (mmHg)** | **Heart rate (bpm)** | **Temperature (°C)** |
| --- | --- | --- | --- | --- | --- | --- | --- |
| **Age** |  |  |  |  |  |  |  |
| <65 years | 1527 | 262 | 1.03 (1.01-1.05)^1^ | 1.07 (1.06-1.09) | 1.00 (0.99-1.00) | 1.01 (1.00-1.02) | 1.25 (1.12-1.41) |
| ≥65 years | 1299 | 225 | 1.04 (1.01-1.06) | 1.03 (1.01-1.05) | 1.00 (0.99-1.00) | 1.00 (0.99-1.01) | 1.20 (1.06-1.36) |
| P for interaction |  |  | 0.075 | 0.000 | 0.496 | 0.013 | 0.492 |
| **Sex** |  |  |  |  |  |  |  |
| Woman | 1070 | 140 | 1.03 (1.00-1.06) | 1.08 (1.06-1.10) | 1.00 (0.99-1.01) | 1.00 (0.99-1.01) | 1.17 (1.00-1.38) |
| Man | 1756 | 347 | 1.03 (1.01-1.05) | 1.04 (1.02-1.06) | 1.00 (0.99-1.00) | 1.00 (1.00-1.01) | 1.23 (1.11-1.36) |
| P for interaction |  |  | 0.538 | 0.110 | 0.747 | 0.854 | 0.869 |
| **Anticoagulants** |  |  |  |  |  |  |  |
| No | 2404 | 399 | 1.03 (1.02-1.05) | 1.06 (1.04-1.07) | 1.00 (0.99-1.00) | 1.00 (1.00-1.01) | 1.29 (1.17-1.42) |
| Yes | 422 | 88 | 1.03 (0.99-1.07) | 1.02 (0.99-1.06) | 1.00 (0.99-1.01) | 1.02 (1.00-1.03) | 0.90 (0.73-1.10) |
| P for interaction |  |  | 0.464 | 0.084 | 0.778 | 0.283 | 0.006 |
| **Antihypertensives** |  |  |  |  |  |  |  |
| No | 2311 | 348 | 1.04 (1.02-1.05) | 1.05 (1.04-1.07) | 1.00 (0.99-1.00) | 1.00 (1.00-1.01) | 1.22 (1.11-1.35) |
| Yes | 515 | 139 | 1.02 (1.00-1.05) | 1.05 (1.02-1.07) | 1.00 (0.99-1.00) | 1.00 (0.99-1.01) | 1.23 (1.04-1.45) |
| P for interaction |  |  | 0.239 | 0.158 | 0.431 | 0.977 | 0.959 |
| **Lipid-lowering drugs** |  |  |  |  |  |  |  |
| No | 2585 | 422 | 1.03 (1.02-1.05) | 1.05 (1.04-1.07) | 1.00 (0.99-1.00) | 1.00 (1.00-1.01) | 1.19 (1.09-1.31) |
| Yes | 241 | 65 | 1.03 (0.99-1.07) | 1.04 (1.00-1.08) | 1.00 (0.99-1.01) | 1.00 (0.98-1.01) | 1.45 (1.14-1.85) |
| P for interaction |  |  | 0.283 | 0.153 | 0.553 | 0.248 | 0.400 |
| **Platelet inhibitors** |  |  |  |  |  |  |  |
| No | 2613 | 436 | 1.03 (1.02-1.05) | 1.05 (1.03-1.06) | 1.00 (0.99-1.00) | 1.00 (1.00-1.01) | 1.21 (1.11-1.33) |
| Yes | 213 | 51 | 1.00 (0.95-1.05) | 1.08 (1.04-1.13) | 1.00 (0.98-1.01) | 1.00 (0.98-1.02) | 1.30 (1.00-1.70) |
| P for interaction |  |  | 0.888 | 0.660 | 0.411 | 0.879 | 0.997 |
| **Oxygen support** |  |  |  |  |  |  |  |
| No | 2612 | 435 | 1.03 (1.02-1.05) | 1.05 (1.03-1.06) | 1.00 (0.99-1.00) | 1.00 (1.00-1.01) | 1.22 (1.11-1.33) |
| Yes | 214 | 52 | 1.03 (0.98-1.08) | 1.07 (0.99-1.17) | 1.00 (0.99-1.02) | 1.01 (0.99-1.03) | 1.19 (0.88-1.61) |
| P for interaction |  |  | 0.164 | 0.645 | 0.183 | 0.284 | 0.725 |

^1^Values are Hazard Ratios (95% Confidence Intervals) adjusted for age, sex (stratified), medications (antihypertensives, anticoagulants, lipid-lowering drugs, and platelet inhibitors), and oxygen support (L/min) at the first vital sign measurement and all vital signs simultaneously.

**Supplemental Table 2:** **Vital signs and the risk of admission and in-hospital mortality by subgroups of potential risk factors of severe COVID-19**

|  | **Number of Participants** | **Number of events** | **Respiratory rate (breaths/min)** | **Saturation (%)** | **Systolic blood pressure (mmHg)** | **Heart rate (bpm)** | **Temperature (°C)** |
| --- | --- | --- | --- | --- | --- | --- | --- |
| **Age** |  |  |  |  |  |  |  |
| <65 years | 1527 | 37 | 1.00 (0.95-1.06)^1^ | 1.03 (0.98-1.08) | 1.01 (0.99-1.03) | 1.01 (0.99-1.03) | 1.03 (0.74-1.43) |
| ≥65 years | 1299 | 259 | 1.05 (1.03-1.07) | 1.01 (1.00-1.03) | 0.99 (0.99-1.00) | 1.00 (0.99-1.00) | 0.97 (0.86-1.09) |
| P for interaction |  |  | 0.596 | 0.751 | 0.061 | 0.202 | 0.695 |
| **Sex** |  |  |  |  |  |  |  |
| Woman | 1070 | 95 | 1.05 (1.01-1.09) | 1.02 (0.98-1.05) | 0.99 (0.98-1.00) | 1.00 (0.99-1.01) | 1.13 (0.93-1.38) |
| Man | 1756 | 201 | 1.03 (1.01-1.06) | 1.02 (1.00-1.04) | 0.99 (0.99-1.00) | 1.00 (0.99-1.01) | 1.02 (1.00-1.04) |
| P for interaction |  |  | 0.293 | 0.831 | 0.511 | 0.970 | 0.104 |
| **Anticoagulants** |  |  |  |  |  |  |  |
| No | 2404 | 246 | 1.04 (1.02-1.06) | 1.02 (1.00-1.04) | 0.99 (0.99-1.00) | 1.00 (0.99-1.01) | 0.98 (0.87-1.11) |
| Yes | 422 | 50 | 1.02 (0.97-1.07) | 1.02 (0.97-1.07) | 0.98 (0.97-1.00) | 1.00 (0.98-1.02) | 1.21 (0.90-1.62) |
| P for interaction |  |  | 0.675 | 0.727 | 0.086 | 0.473 | 0.163 |
| **Antihypertensives** |  |  |  |  |  |  |  |
| No | 2311 | 218 | 1.04 (1.02-1.06) | 1.02 (1.00-1.04) | 0.99 (0.99-1.00) | 1.00 (0.99-1.01) | 1.03 (0.91-1.18) |
| Yes | 515 | 78 | 1.03 (0.99-1.07) | 1.02 (0.98-1.06) | 0.99 (0.98-1.00) | 0.99 (0.98-1.00) | 0.98 (0.77-1.24) |
| P for interaction |  |  | 0.408 | 0.670 | 0.944 | 0.102 | 0.231 |
| **Lipid-lowering drugs** |  |  |  |  |  |  |  |
| No | 2585 | 243 | 1.04 (1.02-1.06) | 1.02 (1.00-1.04) | 0.99 (0.99-1.00) | 1.00 (0.99-1.01) | 1.03 (0.91-1.18) |
| Yes | 241 | 53 | 1.06 (1.00-1.12) | 0.99 (0.95-1.04) | 0.99 (0.98-1.00) | 1.00 (0.98-1.02) | 1.10 (0.83-1.45) |
| P for interaction |  |  | 0.942 | 0.214 | 0.756 | 0.675 | 0.715 |
| **Platelet inhibitors** |  |  |  |  |  |  |  |
| No | 2613 | 259 | 1.04 (1.02-1.06) | 1.03 (1.01-1.05) | 0.99 (0.99-1.00) | 1.00 (0.99-1.01) | 1.06 (0.93-1.19) |
| Yes | 213 | 37 | 1.04 (0.98-1.11) | 0.96 (0.90-1.02) | 0.98 (0.97-1.00) | 0.99 (0.97-1.02) | 0.90 (0.64-1.27) |
| P for interaction |  |  | 0.156 | 0.035 | 0.604 | 0.141 | 0.104 |
| **Oxygen support** |  |  |  |  |  |  |  |
| No | 2612 | 263 | 1.04 (1.02-1.06) | 1.02 (1.00-1.03) | 0.99 (0.99-1.00) | 1.00 (0.99-1.01) | 0.97 (0.86-1.09) |
| Yes | 214 | 33 | 1.03 (0.97-1.08) | 1.14 (1.02-1.27) | 0.99 (0.97-1.01) | 1.00 (0.98-1.03) | 1.83 (1.17-2.86) |
| P for interaction |  |  | 0.630 | 0.028 | 0.516 | 0.305 | 0.075 |

^1^Values are Hazard Ratios (95% Confidence Intervals) adjusted for age, sex (stratified), medications (antihypertensives, anticoagulants, lipid-lowering drugs, and platelet inhibitors), and oxygen support (L/min) at the first vital sign measurement and all vital signs simultaneously.

**Supplementary figure 1**: **Matrix with Pearson correlation test**

***
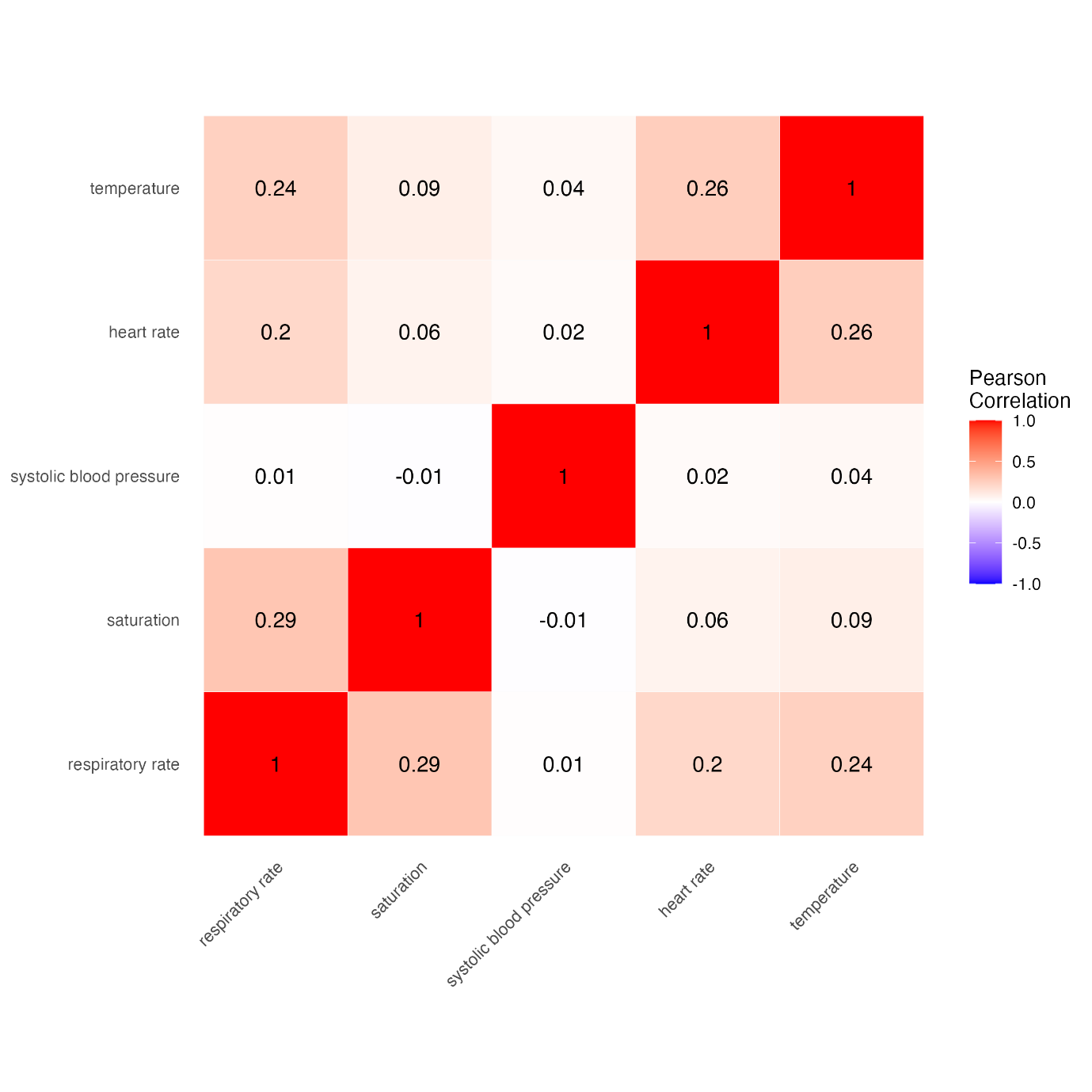
***

The matrix shows that no vital sign pairs had a Pearson value above 0.7
